# Time Development in the Early History of Social Networks: Link Stabilization, Group Dynamics, and Segregation

**DOI:** 10.1371/journal.pone.0112775

**Published:** 2014-11-17

**Authors:** Jesper Bruun, Ian G. Bearden

**Affiliations:** 1 Department of Science Education, University of Copenhagen, Copenhagen, Denmark; 2 Niels Bohr Institute, University of Copenhagen, Copenhagen, Denmark; University of Zaragoza, Spain

## Abstract

Studies of the time development of empirical networks usually investigate late stages where lasting connections have already stabilized. Empirical data on early network history are rare but needed for a better understanding of how social network topology develops in real life. Studying students who are beginning their studies at a university with no or few prior connections to each other offers a unique opportunity to investigate the formation and early development of link patterns and community structure in social networks. During a nine week introductory physics course, first year physics students were asked to identify those with whom they communicated about problem solving in physics during the preceding week. We use these students' self reports to produce time dependent student interaction networks. We investigate these networks to elucidate possible effects of different student attributes in early network formation. Changes in the weekly number of links show that while roughly half of all links change from week to week, students also reestablish a growing number of links as they progress through their first weeks of study. Using the Infomap community detection algorithm, we show that the networks exhibit community structure, and we use non-network student attributes, such as gender and end-of-course grade to characterize communities during their formation. Specifically, we develop a segregation measure and show that students structure themselves according to gender and pre-organized sections (in which students engage in problem solving and laboratory work), but not according to end-of-coure grade. Alluvial diagrams of consecutive weeks' communities show that while student movement between groups are erratic in the beginnning of their studies, they stabilize somewhat towards the end of the course. Taken together, the analyses imply that student interaction networks stabilize quickly and that students establish collaborations based on who is immediately available to them and on observable personal characteristics.

## Introduction

The formation and evolution of (social) networks has been modeled by many researchers, who have investigated theoretical models of mechanisms for producing networks resembling empirical networks [1]–[5]. However, longitudinal network data is rare [6], and it is difficult to obtain network data from the time, 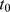, at which the network begins to form. Here, we investigate longitudinal social network data from a time close to 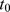.

Students beginning their university studies with few or no prior connections to each other, are in a new situation, and will presumably make new connections with other students as a natural part of their studies. Many of them will also become socially involved, which also involves making new connections to other students. As students become both academically and socially integrated, they may change the ways in which they are connected; such changes might happen on a short time scale, perhaps daily or weekly. Thus, investigating high resolution network data from such students may offer insights as to how such networks form and how they evolve.

If students beginning their studies do not know other students, we could expect them to try out many different possibilities for interaction when they study. Some of these interactions will be deemed worthwhile by the student, and thus continue each week. Other interactions might occur on a less frequent basis and some interactions would only occur once. Another possibility would be that they do not interact much at all about their studies, but mostly work alone. In that case, we would not expect much activity in networks where links depict interactions about study work. If we have additional information about students, for example who they do recitations and laboratory work with, their gender and their grades, we may couple these socially derived attributes with the evolving patterns of connections. These early patterns of interactions in social networks are largely unknown from an empirical point of view.

**Figure 1:**
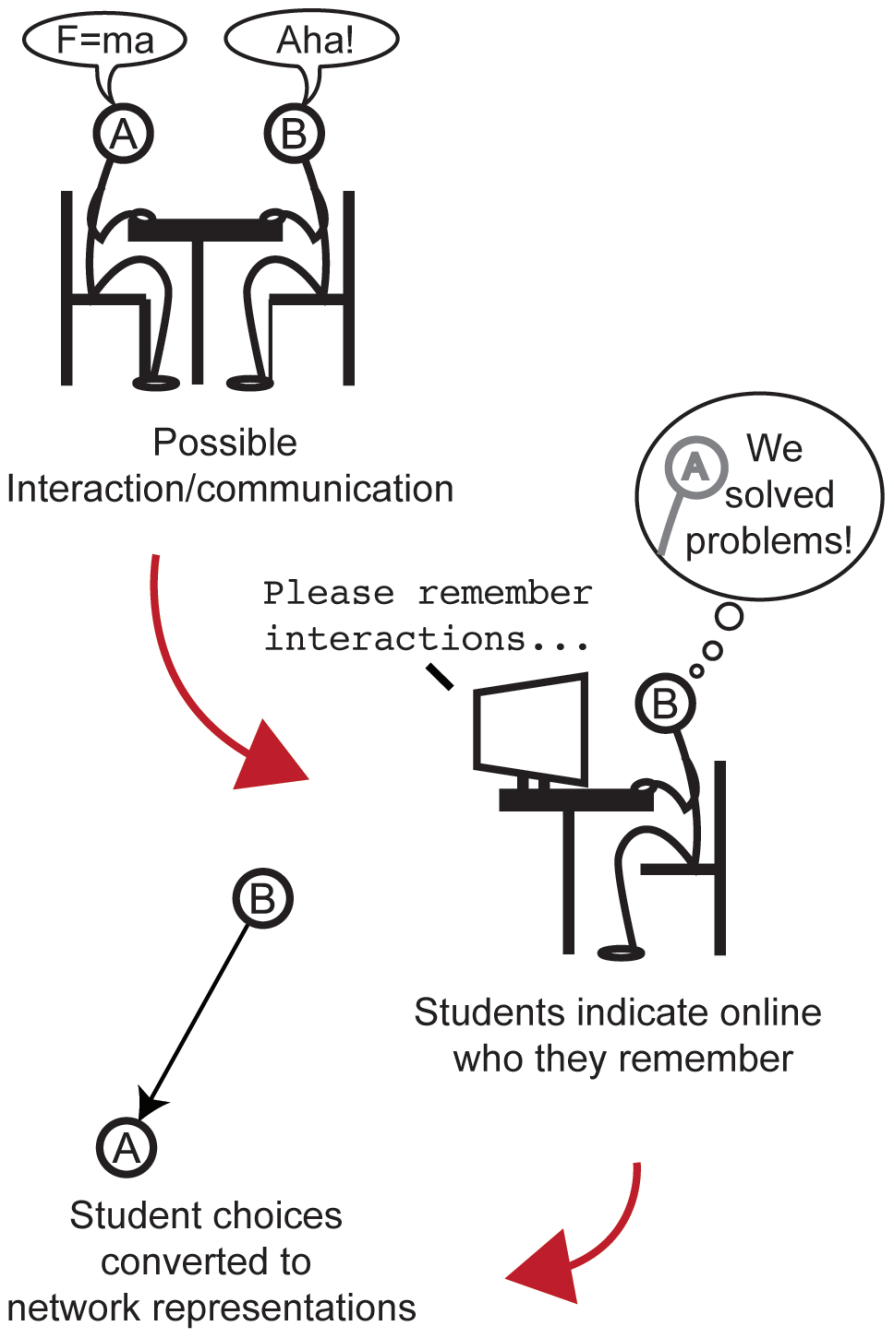
Networks are based on student recall of interactions. Links in these networks are based on the assumption that students remember interactions they had as a part of studying (a). Once a week for nine weeks, sessions were set up so students could indicate the names of fellow students with whom they remembered having communicated about problem solving in physics. Students were asked to consider only the preceding week, and they responded online (b). For each session, the responses were used to create a directed network that models the interactions between students. In each network a directed link exists from a node B to a node A if student B indicated student A in the corresponding session. (c)

This study investigates early interaction patterns among approximately 170 physics students enrolled in an introductory mechanics course at the University of Copenhagen. Students reported recalled interactions about their work in physics on a weekly basis (see Figure 1). Naming another student naturally involves a direction (that is, A “names” B is A to B), so the networks are directed. Self-reported measures are notorious for being biased [2], [7]; however, unlike potentially more objective measures, self-reports can reveal what students are interacting about. Also, using emails, phone calls, or digital proximity as proxies for social ties, may be misleading. For example, it has been found [8] that people remember their friends rather than remembering everyone they are near to as measured by digital means. In contrast, asking students who they remember having communicated with about some subject (in this case, physics), does indicate what the interaction was about. In this work we try to minimize bias [7], [9], [10] by only asking about remembered interactions and not asking students to rank these relations in any way. In particular, we do not ask students to judge the quality of interactions in any way. Recent work [11] suggests that interactions remembered by students are more useful to them than are non-remembered interactions.

To build an understanding of the processes underlying the formation and evolution of social networks, researchers have related network measures to non-network node attributes. For example, for university students the probability of making new social connections has been tied to the number of classes taken together [6]. Also in a 32 yearlong study, people with increasing body mass index (BMI) tend to cluster together [12]. Thus, relating calculations that we can perform on networks to the socially relevant variables can lead to knowledge of social processes relevant to network formation.

In this paper, we characterize weekly networks of student academic interactions through (1) basic properties like degree distributions, plots, and link development (e.g. number of links each week that are new) and (2) community detection and analysis using socially derived attributes to characterize communities. In terms of social networks, finding communities would help an investigator understand the structure of groups in a closed system of people [13], [14]. Communities of nodes are identified by algorithms [15] by optimizing a given quality function [16]. Being directly connected, a property sometimes referred to as homophily [6], is neither a necessary nor sufficient condition for nodes being assigned to the same community. Still, in social networks, the expectation is that nodes in a community share one or more attributes. Thus, it would be interesting to analyze communities found by an algorithm in terms of the distribution of node attribute types within groups. This would give a more detailed understanding of the structure of the network by giving an answer to the question: What binds people together in communities found by community detection algorithms?

The literature is abundant with different community detection algorithms. We focus this study on the Infomap algorithm [17] that has been shown to be both robust and usable for directed networks. Infomap uses a random walker that is able to teleport as a proxy for information flow, and minimizes the description length of a random walk over a set of communities. Infomap is one of the few community detection algorithms that takes directed links into account and it has been shown to perform well on benchmark directed networks compared to other community detection algorithms [15].

Community detection benchmark studies have mostly employed an information theoretical measure called the normalized mutual information to measure the overlap between two community designations. We used the variation of information [18] since it is also applicable to different networks with overlapping nodes. In this study, the variation of information is used as a measure of both Infomap's performance on networks of student interactions and differences between communities detected each week.

To visualize and investigate detailed differences between consecutive weeks, we used the infomap communities to create alluvial diagrams [19]. While alluvial diagrams were originally designed to reveal flow patterns, we recast them to show actual student movement.

Such analyses allow us to investigate how students structure themselves during their first months at university. To quantify how students structure themselves in groups we develop a measure of segregation based on Kullback-Leibler divergence [20]. This measure is applied to each group to see how these groups were segregated compared to the cohort's distribution of grade, gender, and section (see below). Further, by giving each group's segregation a weight proportional to the number of students in it, the segregation for the whole network can be calculated. Thus, the segregation is a measure of how each group and the whole network is structured according to a given attribute, compared to the cohort's distribution.

We invite the interested reader to consult the Methods section at the end of the article for details on any of the methods for analyses above. In Methods, we provide (1) definitions of the basic measures we use, (2) descriptions of Infomap, alluvial diagrams, and variation of information, and (3) a derivation of the segregation.

The rest of this article is structured in the following manner. First, we explain the background for data collection and network preparation. Then, in the Results and Discussion section we first characterize networks of student interaction in physics with degree distributions, average degree, and a between week analyses of links. Then, we show an alluvial diagram and flow maps of networks in the study, and we present the results of the variation of information and segregation analyses. Calculations and results that elaborate on the main findings presented in the Results and Discussion section have been placed in File S1. In the final part of the Results and Discussion section we first sum up and then discuss implications of the results. We suggest that including node attributes is a crucial step towards understanding the meaning of communities in social networks.

## Background, Data Collection, and Preparation of Networks

### Cohort and context

Students were allotted time during the obligatory weekly laboratory exercises to fill out online self-report surveys. Typically, students would fill out the survey at some time during the lab exercise, although some chose to fill it out at home. They were encouraged to fill out the survey at the beginning of a lab class, but some fitted in the survey when a natural break came in their lab activities. Students were told that their answers would be confidential and could not be used by their instructors/lecturers to identify individuals. Participation was not mandatory, although students were encouraged repeatedly to to take part in the study. The students in the course attend four hours of (non-required) lecture per week. Students are assigned to *sections*, of which there are seven, and have the opportunity to attend four hours of *problem solving sessions*. Due to budgetary and space constraints, it is not possible to have the required laboratory exercises concurrently, so these are spread throughout the week, using the same sections of students. These sections are also used in the introductory math course taken by these students. Given this, students who attend all sessions on offer will spend at least 24 hours a week together, with roughly 15 hours of this spent in small (less than 30) sections of students.

We can, therefore, assume that a student answered the survey at the same time of week from one week to another. That is, if student 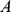 answers the survey on a Tuesday afternoon one week, chances are that student 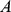 answers the survey on the following Tuesday again. However, students were allowed to switch lab exercise hours, if for some reason they were not able to make it to the scheduled one. Typically, 3 or 4 students per week attended another section. Thus, there is some fuzziness with regards to when student answers are recorded.

The measurements were done during a course in introductory mechanics and special relativity at a large Danish University. Students are primarily ethnic Danes. The majority (roughly 85%) of students are physics majors who have just started their studies. Some major in other disciplines (for example mathematics), but are allowed to choose this physics course as part of their study plans.

### Description of survey and data collection

The online survey was divided into two parts each week; an academic part and a social part. The academic part consisted of 9 interaction categories, while the social part consisted of 3 interaction categories. The categories were developed through a mixed methods pilot project prior to data collection [21], and in this study we only examine the category pertaining to *communication concerning problem solving*. A weekly format was chosen based on [8] who found the greatest correspondence between self-reported networks and digitally measured proximity networks if the interactions were reported within a week. While more finely grained temporal data would be interesting, conversations with a number of students indicate that asking for answers more often than once per week would lead to survey fatigue and significantly lower participation.

Students were given a login to a learning management system, and they were given time during laboratory exercises to take the survey each week. Apart from this, the online nature of the survey made it possible for students to fill it out at any time. For each interaction category, students marked each of the students they remembered having had interactions with. Names of possible students (all students enrolled in the course) were given in a roster [9]. The researcher was present throughout most data collection sessions, and students were invited to ask if they had doubts on how to answer the survey. The researcher emphasized repeatedly that they should mention only the people they remembered, that their answers where anonymous, and that there was no implicit ranking of their friends.

### Preparation of networks

Each week student answers were collected and stored. Due to initial confusion about how to respond, many students did not answer the first two surveys, so the first two weeks' data were combined. Further, due to a technical error, course week 6 data were not recorded. Each of the seven remaining networks was then stripped for self-loops and multiple connections. For each network, we attached three different attributes to nodes: Gender, section, and end-of-course grade.

### Ethics statement

The departments involved (see author affiliations) in the data collection require no specifics ethics submission, and neither has an ethics board nor any formal procedures to be followed in research on human subjects. Instead, an informal agreement with the Heads of Departments was established and discussions with fellow researchers (not involved in this project) and with course instructors were maintained to ensure the ethical treatment of the students. As part of this process, the students were informed orally several times that they would remain anonymous and that their participation was completely voluntary and would have no effect on their course grades. Students who did not want to participate were instructed to simply not answer the survey. The data has subsequently been analyzed anonymously and are secured digitally.

## Results and Discussion

In this section we first give basic characteristics of the networks. We show four networks from four different weeks along with their cumulative degree distribution and the evolution of the distribution of repeated links. To characterize the evolution of links and linking patterns we then show how the links vary across all recorded weeks. Next we show how networks segregate with respect to the three attributes gender, section, and grade, and finally we show the detailed evolution of network groups using an adaption of the alluvial diagram. The technicalities of each of these measures can be found in the methods section. We provide anonymized versions with consistent node labels throughout each of the seven networks. For each network we also provide lists of section, gender, and grade.

### Characterization of networks

**Figure 2:**
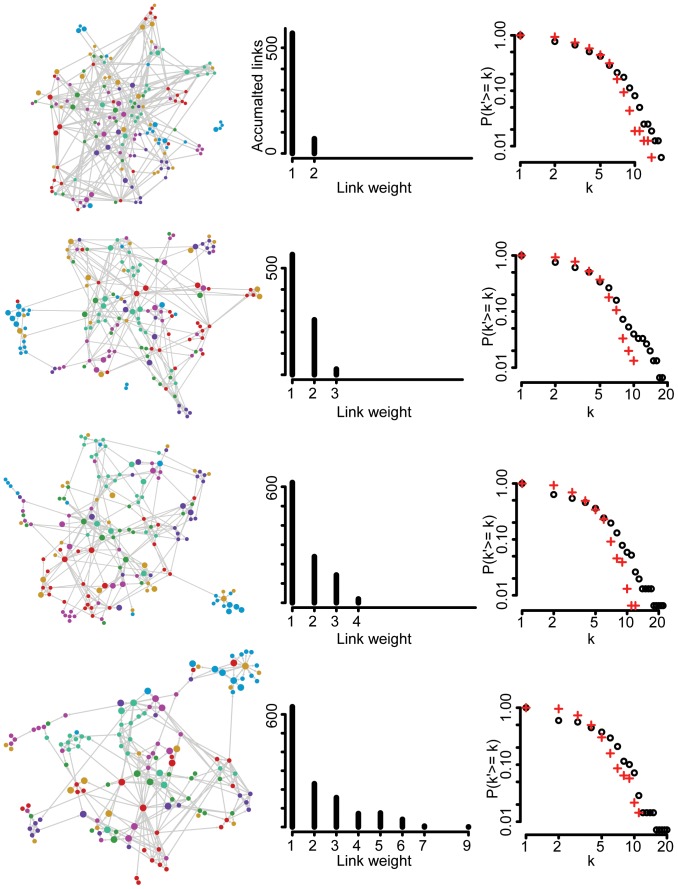
Four networks from different weeks indicate how student interactions develop. The density 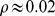 for each network. Colors represent different sections of students. Females are represented by large circles, males by smaller circles. For each week right panel shows the degree distributions (in and out). The middle panels show the accumulated link weight distribution, indicating the extent to which links are reused. The total numbers of nodes in the networks are 161, 152, 154, and 139 respectively.

The introductory course has duration of nine weeks, but for the reasons given above, there are only seven networks, four of which are displayed in Figure 2. The colors the figure denote section number and these are consistent throughout the four networks. For example, if red nodes in the top network belong to section 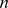, then red nodes in the three other networks also belong to section 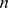. Furthermore, larger circles represent female students, while smaller circles represent male students. Thus, the large grouping on the left of the network representing Course week 4 is composed of students from mainly one section (blue nodes), and five of the students from this section in this grouping are female. Clearly, students form ties across both gender and section, but layout here seems to display at least some clusters of students with an overrepresentation of specific sections and gender. We elaborate on this observation in the Segregation subsection.

**Figure 3:**
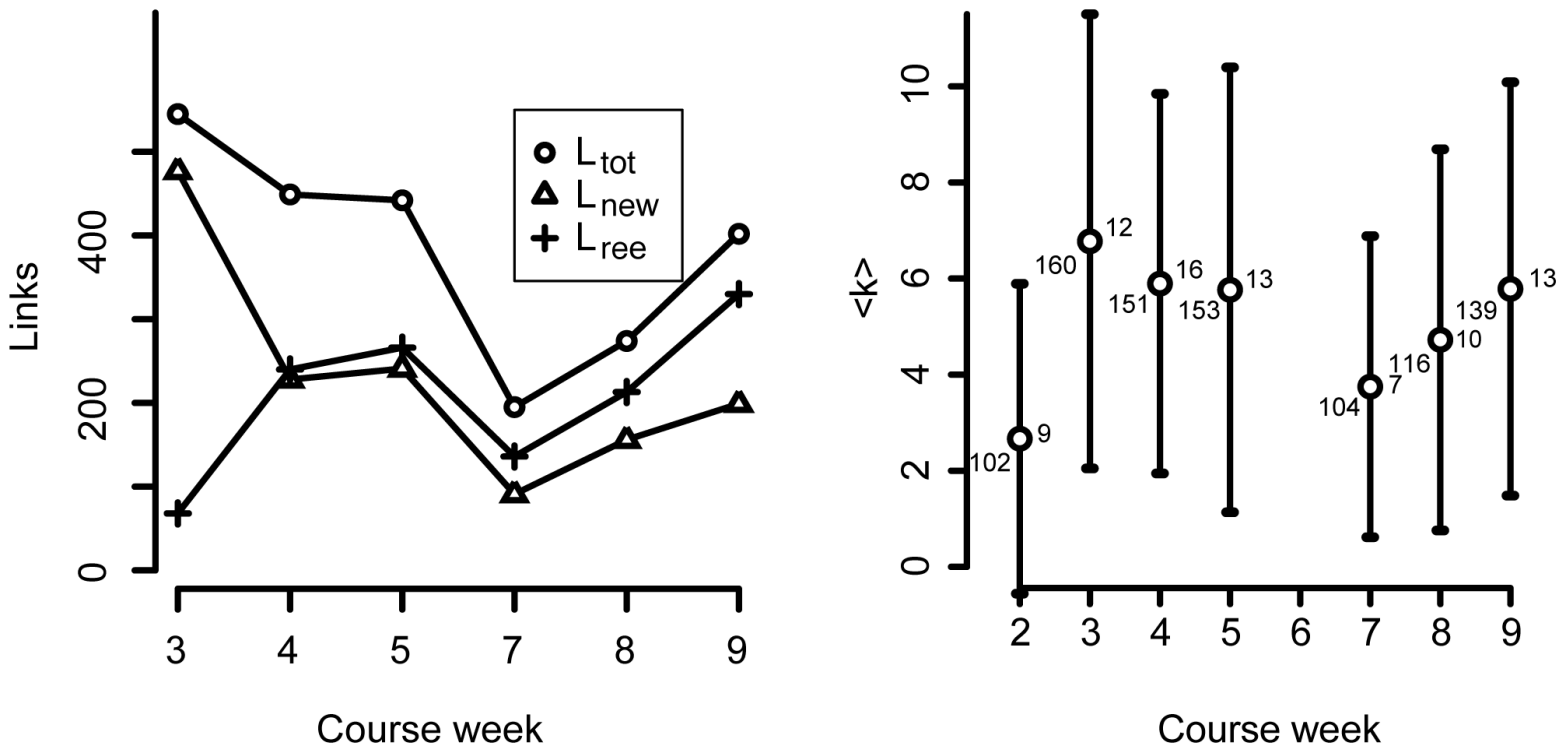
Student interaction networks develop over time. (a) Number of total, new, and reestablished links. New links, 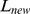, calculated based on the preceding week. 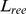 is the number of links in a given week which are also present in at least one of the preceding weeks. 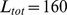 for week 2 (not shown). (b) Average degree with error bars for each week. Numbers at data points indicate the size, 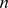, of the network and the diameter.

Turning to the structure of the networks, they seem to evolve from a compact to a more modular nature. This may signify students' tendency to form groups which are connected by bridges [22], [23] as proposed by social network analysis. However, there may also be effects of survey participation since the number of students naming at least one other student for the four weeks is (see also Figure 3b) 124, 114, 97, and 83 for the four networks. This decline in participation could be due to student fatigue with respect to the survey [10], an increased workload on the students towards the end of the course, student drop out (see File S1, where Figure S1 shows that students who turn out to be low-achieving tend to disappear from the network), or to a combination of the three.

The link weight distributions in the middle panes of Figure 2 shows the number of links that re-occur a given number of times. These indicate links that persist throughout the course are rare, but the end of the course most links are re-occurring. The cumulative in-degree (red crosses) and out-degree (black circles) distributions for each network is shown on the right-hand panes. They have sharp cut-offs at around 10 (in-degree) and 20 (out-degree) showing that in these networks is easier to name many people than to be named by many people.

The left-hand side of Figure 3 shows how the total number of links, 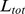 change from week to week. The dip in course week 7 corresponds to the traditional Fall Break in most Danish educational institutions. This dip is peculiar here, since this is an intensive course with no scheduled fall break. However, this could explain both the dip and the slow recovery in course week 8. In week 7, a larger number of students would be absent not participate, and in week 8, fewer people would list having had interactions with these students.

The right-hand shows the average degree of each network with error bars. The size of the error bars indicate broad distributions consistent with Figure 2. The numbers on the left of each data point represent then number of nodes in the network, while the numbers to the right indicate the diameter of each directed network.

**Figure 4:**
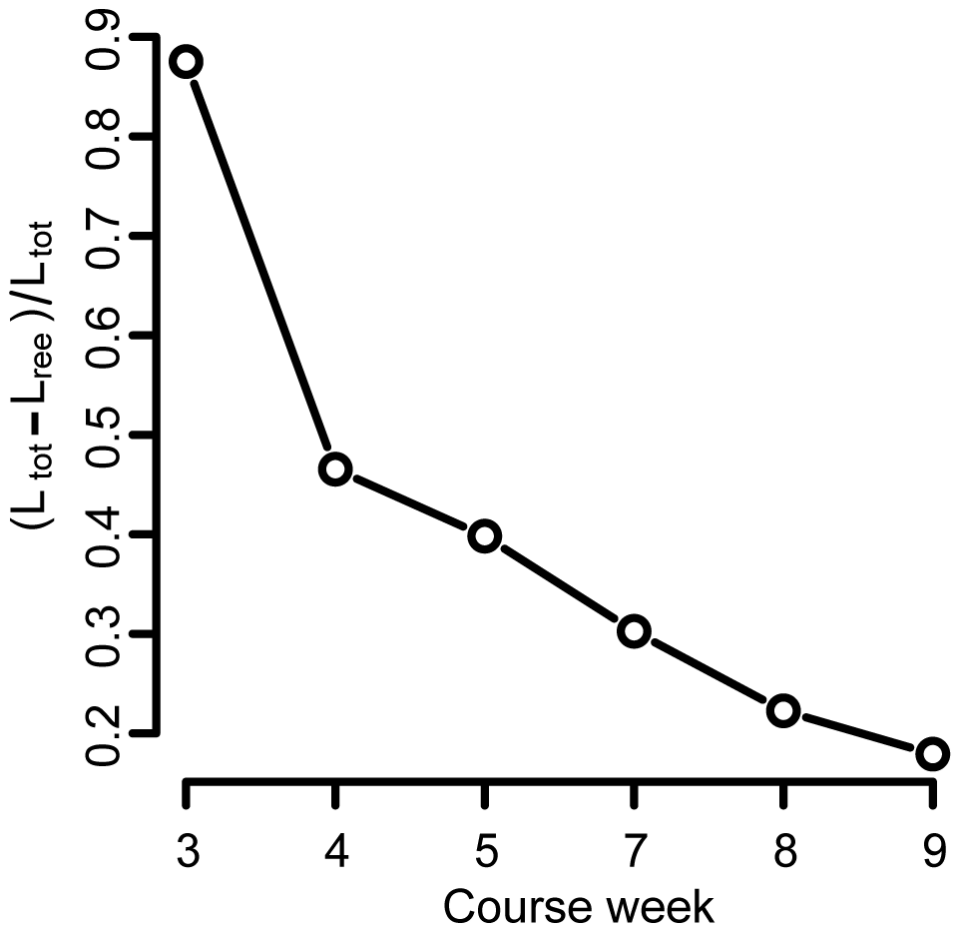
The fraction of completely new links relative to the total number of links, 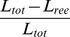, decreases over time. This is not due to students saturating connections, since summed over all weeks; students use a total of 5% of all available links.

There are a considerable number of new links, 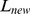, each week compared with the preceding week. Roughly half of the links each week are new compared to the preceding week. However, the number of re-established links, 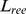, comprise a larger and larger fraction of the total. For a given week, the number of re-established links is the number of links in the network which are present in at least one of the preceding weeks' networks. Together, the variations in 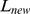 and 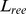 may be used to form a hypothesis about how bonds are created during the early stages of this particular student network's history: students try working with a lot of different collaborators. As they do this, they find out who they want to work with and return to them again. This is supported by Figure 4, where the fraction of completely new links, 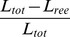, is shown to decrease over time. The number of unique links for all weeks is 1214, which is roughly 5% of the total number of possible links (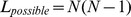) in a directed network with 140–160 nodes (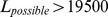)). This implies that the decrease in completely new links is not due to a saturation of the network links.

### Community detection and segregation

We used Infomap [17], [24] to calculate clusters on each of the seven networks. For each network and for each algorithm, we calculated the variation of information, or information theoretical distance [18], between two successive runs. We repeated this procedure 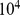 times for each network. Infomap also returned the number of communities found, modularity, and the average per step coding length (see Methods section) for each run of each algorithm. Table 1 shows the average values and standard error for each of these measures. The variation of information (VI) varies between 0 (with no uncertainty) and 0.2(1) bits. This is small compared to both distances between consecutive weeks (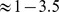 bits) and smaller than the average for a reference modularity optimization algorithm [16] (See Table S1 in File S1).

**Table 1:** Infomap results and performance.

Week	2	3	4	5	7	8	9
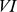	0	0	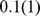	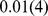	0	0	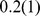
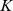	7	28	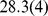	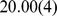	15	17	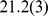
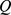	0.15	0.555	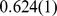	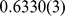	0.725	0.673	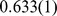
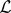	6.77	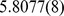	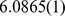	6.3530		6.8210	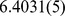

The results of multiple runs of Infomap on each week. 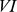 is the average variation of information between two consecutive runs, 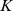 is the average number of communities, 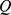 is the average moduluraty, and 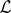 is the average description length found for 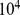 runs.

**Figure 5:**
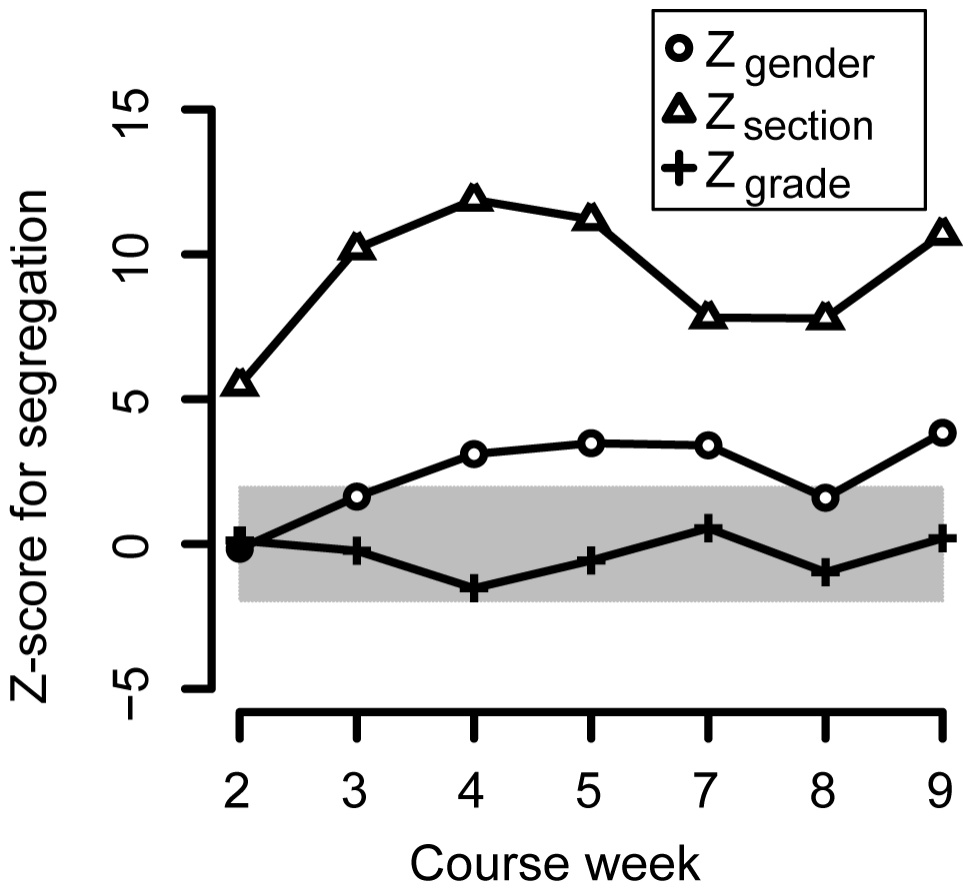
Segregation Z-scores for gender, grade, and section show different behaviors. The shaded area indicates the non-significant region, where 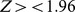. Students segregate according to section, and somewhat according to gender but not according to end-of-course grade.

We calculated the total segregation for each of the section, grade, and gender attributes. The results of the calculations for the whole network segregation from week to week during the course are shown in Figure 5. When 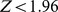, the segregation is indistinguishable from random variation. The expected distribution 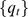 is calculated from the students that are present in the network in a given week. The first week shows neither significant segregation nor non-segregation. During the following weeks, students segregate significantly according to lab classes and to a lesser degree according to gender.

While there is significant segregation according to gender and section, students do not segregate or mix near perfectly (see Methods section). If students segregated perfectly, calculations show that the Z-scores would be around 20 for gender and around 40 for grade and lab class. If they did not segregate at all, that is if 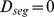 corresponding to perfect mixing, the Z-scores would be around -2 for gender and -4 for grade and lab class. Thus, groups do not consist for example of students from only one lab class but of clusters of students from different classes.

### Development of group structure

**Figure 6:**
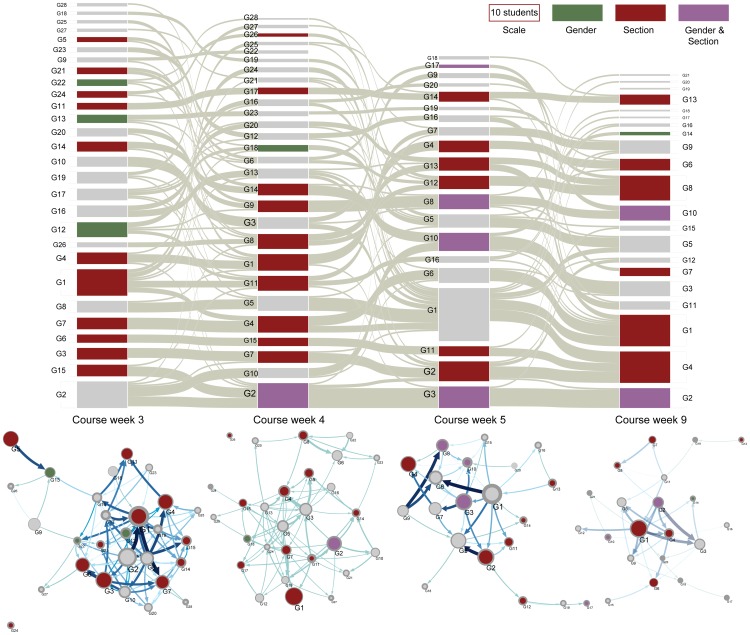
Large scale structures show between-week student movement and within-week information flow. Top: Alluvial diagram for communities of the four networks displayed in Figure 2. The height of a block indicates the number of students in the community (see scale). The thicknesses of the gray streamlines between groups in different weeks indicate between group movements; thicker lines indicate that more students moved together. The color of a box representing a community indicates whether it is significantly segregated (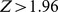) with respect to the given attribute. Bottom: Maps of community structure for the same networks. Node sizes are proportional to accumulated flow rate for a particular community. Labels on the map correspond to labels in the alluvial diagram, allowing for comparison between community size and flow. Arrow sizes are proportional to the information flow between groups as calculated by Infomap. Color codes in the maps have the same meaning as in the alluvial diagram. The total number of communities each week is 28, 28, 22, and 20, respectively.

The top panel of figure 6 shows an alluvial diagram [19] for student groups in the four networks displayed in Figure 2. Each box corresponds to a group of students. The height of each is box is proportional to the number of students in that group (see also the scale). The color of the boxes mark if the groups are significantly segregated (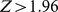) with respect to gender (green), section (dark red) or both (purple).

The streamlines between each column indicate student movements from one week to the other. From course week 3 to 4 there are 71 streamlines indicating 71 movements of varying student sizes. From course week 4 to 5 this number drops to 51 and form course week 5 to 9 even further to 44. Also, average thickness of streamlines seems to become larger, indicating that more and more students move together. This intuitive pattern is confirmed by calculations of the distance between community structure as calculated by the variation of information [18] (see Methods section), which becomes increasingly smaller from week to week (see File S1).

The visualization of segregation gives insights into the composition of individual communities. Many communities are not significantly segregated, which may in part explain why the networks as a whole never reach the maximum segregation for any measure. Moreover, we see that most groups that are significantly segregated with regards to gender are also significantly segregated with regards to section. Thus, it seems that students primarily find their collaboration partners within their section and secondarily within their gender. However, based on the visualization, we would not expect the total segregation of networks to be close to the maximum (see Methods section).

A map of information flow between communities is shown at the bottom for each week. Communities are labelled by a number both in the alluvial diagram and in the flow map, and the colors again signify segregation. Thus, G1 in course week 3 is represented both as a box in the first column of the alluvial diagram and in the flow map below the box. The arrows indicate probability flow, which we can relate to how many students in one community name students in another community. Since a naming indicates communication about problem solving, these arrows might indicate which communities are important for how problem solving knowledge is spread in the network. As such, they might provide an indication of which students need and which students can give help in an introductory physics course. It is worth noticing that large communities do not necessarily have a correspondingly large accumulated flow. The most prominent example is G2 in course week 5. The community contains 32 students, but as can be gathered from the map below the column, the accumulated flow is comparable to much smaller communities. This example is supported by standard linear modelling of size versus flow, which shows that 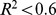 (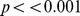). Thus, even though there is a clear accumulation of flow due to community size, large size is not a guarantee for large flow.

### Summary and implications

This study investigated the early stages of network formation based on student self-reports of whom they remembered having communicated with about problem solving in physics. The network data consisted of seven networks made from weekly reports these types of communication in an introductory physics course. We employed several different types of analysis, including both network data and student attribute data, to investigate the structure and nature of these networks.

The link analysis showed that roughly half of the links in a week were new compared with the preceding week. However, as weeks go by, students communicate with former communication partners. This is indicated by the relative decrease in the total number of completely new links. One interpretation of this result is that students try out many different collaboration partners throughout course weeks and maintain collaborations that they find valuable. By construction, the nature of collaborations in these networks has to do with problem solving in physics, so the results yield information about pathways by which ways of solving problems in physics spread amongst students. Students who collaborate often have more opportunities to influence on each other's problem solving techniques than students who collaborate only once. One question for future research on networks is whether these opportunities are used or not.

Stream lines in alluvial diagrams showed that while students shift between communities from week to week, the tendency is that communities stabilize over the nine weeks of this physics course. This is consistent with the link analysis, but it also expands the picture. Since students in a community need not be directly connected to be grouped we could use the communities as indicative of connections that are not reported by students. Since self-reports are biased in a number of ways, for example, by humans' ability to recall interactions, researchers may always question the reliability of the network created in this way. For example, if we were able to objectively record students' real-time behavior, we could reasonably expect students that were identified as a community in the problem solving network to also be near each other during study activities pertaining to problem solving.

The student segregation analysis of the communities found by Infomap showed that students segregate significantly (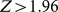) to according to section number and to lesser extent gender, but at no point according to grade. This seems to indicate that when new networks are formed, people tend to group themselves based on immediate availability and observable personal characteristics (e.g. gender). Since people were randomly distributed according to section number, there is no reason to believe that this should have any other effect that to make random people available to each other for collaborations. Still, this is the most significant factor for segregation. A person being either male or female is a superficial sign, since in most cases it is easy to distinguish between the two. Also, we might expect (fe)males to on average feel similar to other (fe)males. Then, significant gender segregation across weeks implies that recognizing another person as being similar to one self, might form the basis of collaboration. Grades are not easily accessible signs or they may not be a valid similarity measure, and this could explain why people do not segregate according to them. As an attribute, grade seems to be indicative of other processes than homophily. As is shown in Figure S1 (File S1), students with low or failing grades become less prevalent in the network towards the end of the course.

This study shows that it is possible to characterize communities found with community detection algorithms in terms of node attributes. A possible extension of this study would be to ask students if they recognize the communities detected by the algorithm. If a student is clustered together with another student without them being directly linked in the corresponding network, will they then recognize each other? Will non-reciprocal links be recognized as reciprocal? If the answer is “yes”, then one could argue that clustering algorithms can help detect communities from incomplete data. Another set of questions are related to the segregation: Do students recognize that grade is unimportant for clustering? And that section is? Do they recognize the gender segregation? On the long term, will they group more according to grade and gender? Or will the original section follow them throughout their studies?

## Methods

Unless otherwise stated calculations have been done within the R package for statistical programming [25] using the igraph package [26]. All R-functions, anonymized data files, and node attributes used in this study are available in File S2.

### Basic characterization of networks

#### Visualization

We visualize four of the networks using software Gephi [27] and the Force Atlas 2 algorithm [28]. We color the nodes according to external attributes. In the results, we color the nodes according to section. In Figure S1 (File S1) we show the four networks colored with respect to grade. We use large circles to denote females and small circles to denote males.

#### Link weight distribution

Some connections are re-established in subsequent weeks. The link weight distributions shows in a cumulative manner, how many students have named another student once, twice, thrice, etcetera. The final link weight distribution integrates all networks in the study. Formally, link weights for course week 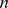 is 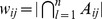, where 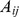 is the adjacency matrix.

#### Cumulative degree distribution and average degree

We use the degree sequences to generate cumulative degree distributions, average degree and standard error on the average degree. First, define 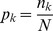, where 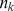 is the number of nodes with degree (in, out, or total) 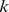. The cumulative degree distribution is then [13]: 
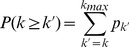



We calculate the m'th moment as 
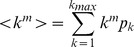



For each network, we use this to calculate 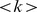 and the standard deviation as 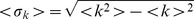



#### Link development

To characterize link development throughout the period, we define three different kinds of links:

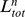
 is the total number of links in network for a week 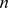.

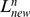
 is the number of links in network for a week which were not in a graph for the previous week. Formally, if 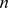 refers to the network for a given week, 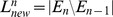.

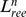
 is the number of links in the network for week 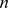, which were present in any one preceding week. Formally 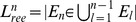

Together these measures characterize the variation of links. 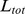 can be seen as a measure of the total activity in the network. A higher value then signifies more interactions. 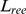 measures the degree to which students tend to re-connect at a later point in time. A growing value signifies that people tend to interact more with people they already know. 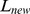 measures how much connections fluctuate from week to week. A high value means that people tend to interact with different people from one week to the other. However, 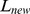 has no memory of other weeks.

### Community detection and analysis

#### Community detection with Infomap

We have used Infomap [17] to detect communities on networks. Infomap is based on the hypothesis that communities in networks can be detected as a set of local structures that minimize the information cost of describing paths through networks. Seen in this way, the function of communities is to make it easier to keep track of information about the network.

One can consider a network of 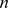 nodes as a codebook with 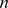 words. To simulate information flow, Infomap uses a random walker that visit nodes using links. During a walk, node 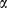 is visited a fraction 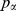 of the time. This would correspond to using the 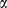
'th word a fraction 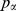 of the time. In Huffman coding one would then assign each node a code word with length proportional to the visit frequency, 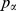. With such a scheme, the expectation value of the minimal amount of code needed to describe a single step in the random walk is 
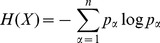
(1)


Infomap introduces the possibility of making several codebooks on the network, where each codebook, 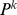, represents a community, 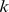. The advantage of this approach is that one can reuse code words in different codebooks. One then has to keep track of each of the new codebooks, 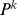, with an index codebook, 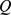. The core principle in Infomap is to join nodes into a set of 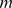 communities, called the partitioning 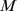. This partitioning is described by

1. An index codebook, 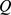, that describes the network on a community level. The random walker will change from community 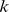 a fraction 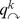 of the time. Relative to other communities' codebooks, community 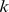
's codebook is then used at a rate of 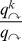, where 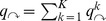 Thus, assigning code words to communities in an optimal way yields an expected information cost of 
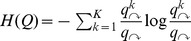
 for using the index codebook.
2. A set of 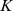 different codebooks over nodes, 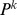, each with an expected information cost of 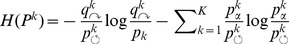. Here, 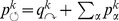. The 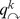
's are needed in the community codebook to ensure that we keep track of when the random walker leaves the community. Thus, each community needs to allow for one extra code word, an exit code, on top of the code words needed for each of the nodes.

The index codebook is needed a fraction 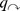 of the time; when the walker changes module. Thus the expected contribution from using the index codebook is 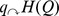. Likewise, each community codebook is only used when the walker is in the community (or exiting the community), and this happens with a frequency 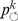. Thus the contribution from each community codebook to the total expected information cost is 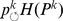. For a given partitioning, 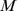, of a network, then, the expected per step code length must then be the weighted sum of all these 
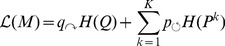
(2)


The Infomap algorithm joins nodes to communities and then allows sub-communities and single nodes to move between communities until the combined information cost is minimized. The details of the minimization is given in [24].

Infomap has been modified several times since it was first introduced [29]–[31], including different teleportation and walk-recording procedures, multilevel code books, overlapping community structure. While these changes and other methods [15] for identifying clusters could yield interesting differences, in this work we report on the original Infomap as an example of a robust community detection algorithm that is applicable for directed networks.

#### Alluvial diagrams to visualize transitions of students

Alluvial diagrams can be used to map changes in networks [19]. The idea is to compare network partitionings with overlapping nodes at two or more points in time. Originally, communities' accumulated flow was shown and compared, and stream lines connected groups to represent changes in accumulated flow. Thus, in a social network, a community with a small flow rate would be shown as small in the alluvial diagram even if the community contains a large number of nodes. In contrast, we use the alluvial diagrams to portray the size of communities, not their flow.

Starting from networks 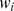 and 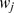 for two consecutive course weeks, we used Infomap to create partitionings 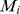 and 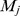. The partitioning 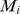 contains 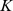 communities 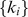 with sizes 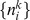, where 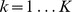. Likewise, 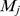 contains 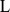 communities 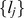 with sizes 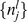, where 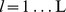 In the alluvial diagram each community is shown as a box with height 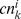, where 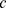 is a scaling factor. Streamlines that connect community 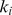 with community 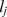 represent the number of students that were placed in 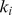 and in 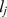. Thus, in this alluvial diagram streamlines are a visualization of the confusion matrix used to calculate the variation of information between partitionings [18] (see next section).

As a further visualization, we assign color to communities based on their segregation score (see section Node segregation measure). We use red for groups that are significantly segregated (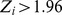) with respect to section, green for groups that are significantly segregated with respect to gender and purple for groups that are segregated with respect to both.

Interested readers may consult Figure S2 in File S1 to see alluvial diagrams based on the original [19] flow rate based idea.

#### Information based distance between partitionings

To characterize the difference between two partitions of nodes, we use the variation of information (VI) between the two partitions [18]. Given two partitionings 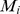 and 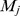 of 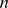 nodes, VI is calculated by constructing the confusion matrix, 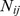. If 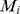 has 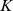 modules and 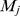 has 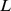 modules, the dimensions of the confusion matrix is 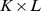. The 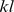
'th element of 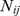 is equal to the number of nodes, 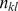 in the k'th module in 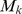 that are also in the l'th module in 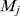. Given the three probability distributions for 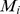, 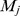 and their overlap, 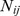, 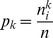, 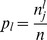, and 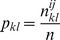, the entropies of these distributions, 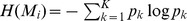, 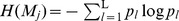, 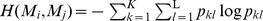 can be calculated. The variation of information can then be calculated as: 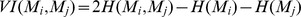
VI is a metric [18], which means that the numbers obtained can be intuitively understood as distances. For each network the distance cannot be more than 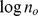, where 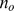 is the number of overlapping nodes. Since the number of overlapping nodes vary between 102 and 160, 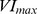 varies between 6.67 and 7.32.

In this work we use this measure to characterize the stability of Infomap (directed) as applied to the networks of this study. In File S1 and File S2 we have compared Infomap undirected with undirected simulated annealing.

Since networks for different weeks will have an overlap of nodes, we can also use VI as a measure of the distance between partitionings of different weeks. Since the method is built upon the confusion matrix, VI only considers nodes that are common to both networks into account. Thus we use reduced groupings, when we compare between weeks, but otherwise the procedure is the same as described above. Measuring the difference between consecutive weeks allow us to describe how groups stabilize over time quantitatively. A smaller distance between groupings, indicate that students tend to stay more in the same groups than if the distance is larger. The detailed results of these calculations are given in File S1 and File S2.

### Node segregation measure

Here, we develop measure of node segregation: The degree to which nodes are partitioned into communities with similar nodes. The measure can be used if nodes can be partitioned categories based on an attribute. For example, gender would be an attribute with two categories, male and female. Given a partitioning, 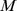, that contains 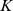 communities and given that each node belongs to one of 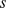 different categories describing an attribute, we seek a number that tells us the degree to which nodes in a community 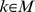 are the same. Here, we first illustrate the measure when the number of attribute categories 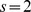, so that each node either has the value 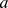 or the value 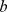. We then generalize to an arbitrary number of attribute categories.

Each group 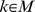 consists of 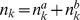 nodes. Thus, in group 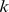, the probability of choosing a node at random with the value 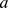 is 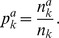
However, if we choose a node at random from the entire network, we have to consider all nodes in the network, and then 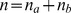. Thus, the probability, 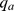, of picking one with the value of 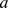 is 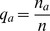



In information theory, the cross-surprisal [20] is the information gained relative to the information known prior to the measurement is 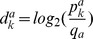. Taking the expectation value for the 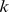
'th group yields the cross-entropy or Kullback-Leibler divergence [20] for that group: 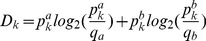
(3)


For the 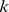
'th group, (3) expresses the expected information gain relative to what was known before. For the partitioning 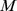, we now require that it does not depend on the 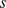 categorical variables. That is, we assume that the distribution over the categorical variables in one group is independent of the distribution in another group. Then the cross-entropy is additive and each group contributes to the total weighted cross-entropy of the system given the partition 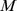 in proportion to its size: 
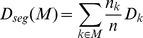
(4)


In general, attribute categories may divide node in more than 2 different categories. We now consider 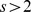. The probability of selecting a node from the 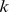
'th group with the 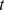
'th attribute category is 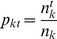
where 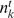 is the number of nodes in the 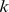
'th group with the 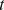
'th attribute. Thus, the total number of nodes in the 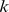
'th group is 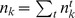. Similar to the situation before, selecting nodes at random from the network, we expect a probability distribution 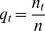
with 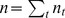. Then the cross-entropy for the 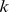
'th group is 
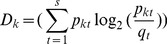
(5)The total weighted cross-entropy of segregation is then:
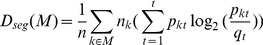
(6)


The range of 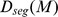 can be determined as follows: If 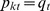 for all 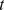 in all groups in 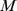, 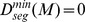



For perfect segregation, a group will only contain nodes with a particular attribute 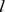 of the 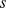 attributes. Thus, 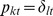 (where 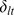 is the Kronecker-delta) for the 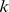
'th group. Then (5) reduces to 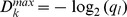. The segregation for the entire network becomes: 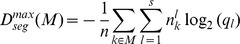
Setting 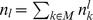, collecting terms of 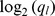, and using 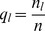, perfect segregation, 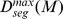, reduces to the Shannon entropy of the 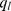-distribution: 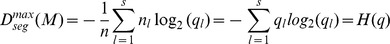
The final step is to calculate how different the network's segregation is from random variation. We adopt the Z-score [32] to this purpose: First, the attributes are randomly re-distributed on the nodes, while keeping the network structure and partitioning 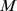. In this study, we make 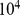 random re-distributions like this. For each random redistribution, we calculate 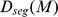. Calculating the mean and standard deviation over the randomized samples, the Z-score becomes:
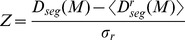



In this study, the segregation Z-scores of different three different attributes are calculated: Gender (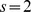), grade (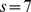), and lab class (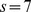).

Notice that we can also measure community wise Z-scores by using (3) and following the randomization procedure described above. Then it is possible to see which groups contribute to the segregation, and one can scrutinize the structure and composition of the group. We use the community wise measure in Figure (6) to show groups that are significantly (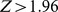) segregated. Detailed results of these calculations are available in Tables S3-S6 in File S1.

## Supporting Information

File S1
**Auxiliary calculations and visualizations.** Variation of information, 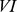, calculations, visualizations based on grade, and further discussions of these calculations and visualizations.(PDF)

File S2 **Anonymized data, node attributes, and R-functions.** Unweighted, directed networks, with consistent node id's for all course weeks, attributes for each node in each network, and R-functions developed for this study.
(ZIP)
