# Supplementary material for: Time Development in the Early History of Social Networks: Link Stabilization, Group Dynamics, and Segregation
Source: PLoS One. 2014 Nov 17;9(11):e112775. doi: 10.1371/journal.pone.0112775 (PMC4234624; doi:10.1371/journal.pone.0112775)
Supplement: File S1 — Auxiliary calculations and visualizations. Variation of information, , calculations, visualizations based on grade, and further discussions of these calculations and visualizations. (PDF) [file pone.0112775.s001.pdf]

File S1: Auxiliary calculations and visualizations

Jesper Bruun[1], Ian G. Bearden[1,2]

1 Department of Science Education, University of Copenhagen, Copenhagen, Denmark

2 Niels Bohr Institute, University of Copenhagen, Copenhagen, Denmark

∗ E-mail: jbruun@ind.ku.dk

We report on auxiliary calculations and show visualizations that are alternative to the main article's visualizations. First we visualize the four networks shown in the main article's Figure 2 from a different perspective. We color them according to end-of-course grades rather than section. This does not show any segregation, but it does yield information about the students that end up as poor performers. This section is followed by an alluvial diagram which shows community grades and flow rates rather than segregation and student movement as in the main article. Next, we show results from VI calculations on two different community detection algorithms, and we compare these calculations with Table 1 in the main article. We then calculate the between week VI, and results support the claim that communities stabilize over course weeks. We investigate a linear model of community size versus community accumulated flow rate and find that the model should be improved. Finally, we show detailed results of the community-wise segregation results.

Visualization of networks with grades

In this section, we show networks for course week 3, 4, 5, and 9, corresponding to the networks in Figure 2 of the main article. See figure legend for an explanation of color codes, histograms, and node shapes. As is also evident from calculations using the segregation measure, these networks do not indicate segregation according to the end-of-course grade earned in the course. The non-passing students (red and orange nodes) seem to move from being well integrated in the network to the periphery in week 5 and most red nodes are gone in week 9. This may be explained by the structure of the course, where students are continuously solving problems and getting grades. Students will know early on how well they perform. Thus, students who do poorly might simply have left the course. On the other hand, they might be pursuing the course without participating in the survey or being named by others.

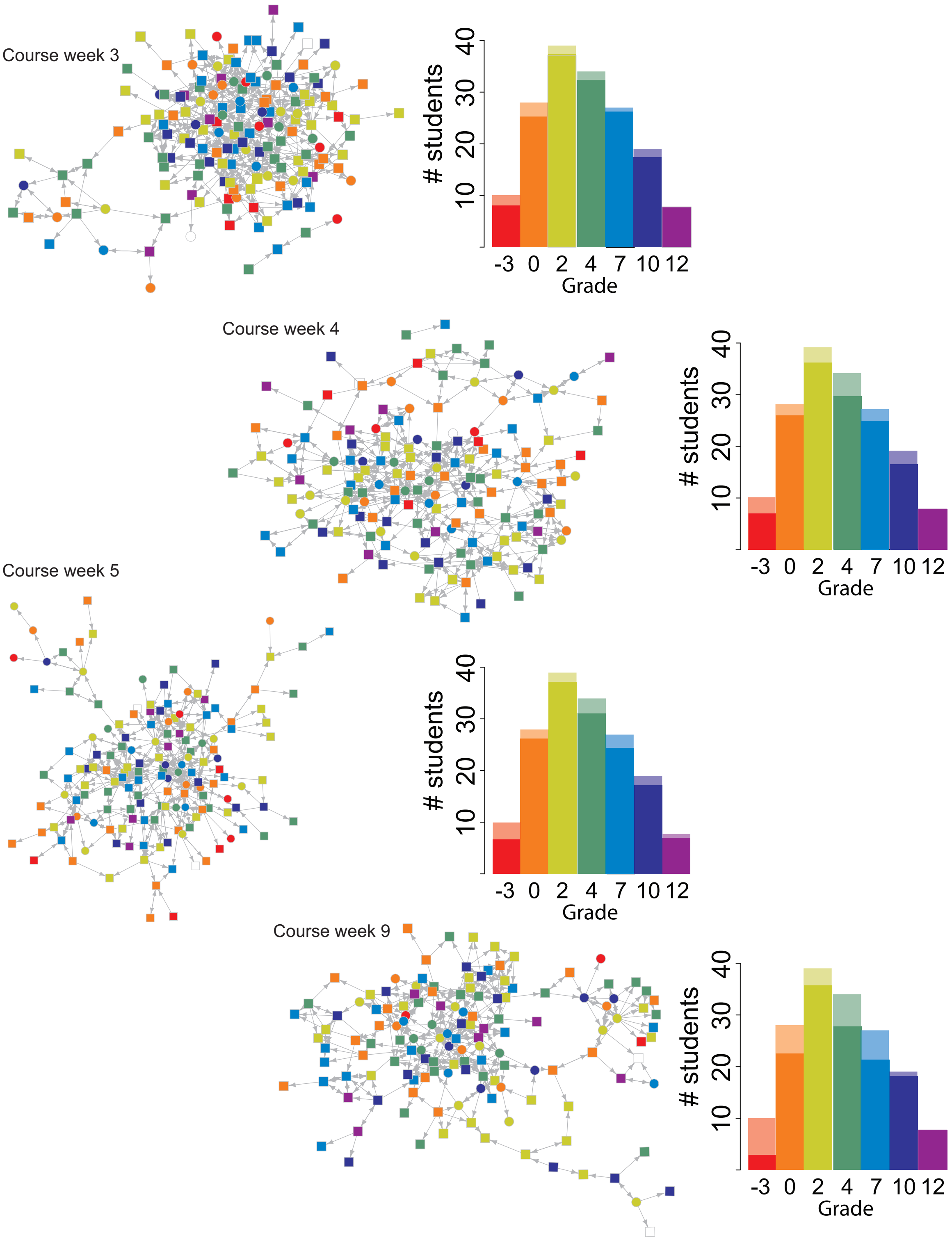

Figure S1. Four networks for different course weeks displayed using a standard force based algorithm. Square nodes represent males and circular nodes represent females. Colors indicate end-of-course grade following the same scheme as the histograms on the right of each network. In these histograms, the number of students with a particular grade is displayed in full color a low opacity background that shows the histogram for all students that followed the course and got a grade. Thus the histograms yield information about how many students that end up with a particular grade are also present in the network.

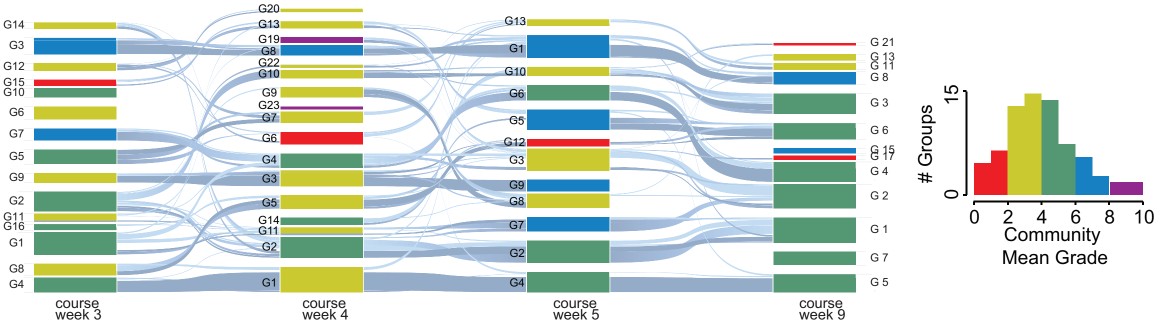

Figure S2. An alluvial diagram using the orginial idea where box size and stream lines are proportional to flow rates. Furthermore, in this representation, box colors represent average grade (see histogram). Groups seem to converge towards the same average grade, which is consistent with the segregation results.

Alluvial diagram with flow-rate and grades

Figure S2 shows the alluvial diagram [1] for student communities in the four networks displayed in Figure S1. The height of each is box proportional to the accumulated flow rate of the community. The color of the boxes mark what range the community mean end-of-course grade falls in. Though there does not seem to be a connection between community mean grade and accumulated flow rate, the community mean grades seem to become more homogeneously distributed among the communities in the diagram in course week 9 compared with the preceding weeks. The histogram on the right shows the community mean grade for all communities found in the four networks.

The streamlines between each column indicates shifts in flow rate from one week to the other. Some groups seem stable throughout the course, but many changes happen between weeks. However, there are fewer stream lines between week 5 and 9 (38) than between the other weeks (46 and 51 respectively), which is consistent with our general finding that communities stabilize somewhat over time.

Comparing Algorithms

To compare Infomaps performance on the directed networks of this study with an modularity optimization algorithm, we performed the same types of calculations as listed in Table 1 in the main article with igraphs spin-glass optimization procedure [2], which we call Spinglass. Results are shown in Table S1. Like most community detection algorithms in igraph, Spinglass disregards directionality of links. While a method for applying modularity optimization on directed networks has been developed [3], it has not yet been

implemented in igraph, and we have not pursued it here. However, future work that seeks to compare the information based approach with the modularity approach might consider this. The results show

Table S1. Undirected Spinglass results and performance

Course Week	2	3	4	5	7	8	9
VI	0.8(3)	0.8(3)	0.7(3)	0.7(2)	0.2(1)	0.4(2)	0.5(3)
K	10.3(6)	11.4(6)	10.7(7)	12.5(8)	8.4(4)	8.8(5)	10.5(5)
Q	0.577(2)	0.459(2)	0.554(2)	0.520(2)	0.570(2)	0.541(1)	0.556(2)

The results of multiple runs of Spinglass on each week treating each week as an undirected network. VI is the average variation of information between two consecutive runs, K is the average number of communities, and Q is the average moduluraty found for 10^4 runs.

that the directed Infomap is much more reliable, produces finer grained maps and better modularity than Spinglass. Reliability follows from the fact that Infomap produces a consistently and significantly smaller VI than Spinglass for consecutive runs of the algorithm. Infomap produces a significantly larger average number of communities, thus yielding finer grained maps of the community structure. Finally - and interestingly - Infomap produces partitionings with higher modularity. The exception is course week 2, where Spinglass has a higher modularity and number of communities.

We also did the calculations with the undirected version of Infomap. The differences to Spinglass are the same as for the directed version of Infomap. Disregarding link directionality makes the average description lenght shorter for most networks.

Table S2. Undirected Infomap results and performance

Course Week	2	3	4	5	7	8	9
VI	0	0.1(1)	0.2(1)	0.00(1)	0	0	0.1(1)
K	7	28	21.9(6)	19	15	17	18.00(3)
Q	0.15	0.5815(9)	0.658(2)	0.657(0)	0.746	0.680	0.6630(3)
$\mathcal{L}$	4.694	5.833(1)	5.484(1)	5.480(0)	4.507	4.959	5.376(0)

The results of multiple runs of Infomap on each week treating each week as an undirected network. VI is the average variation of information between two consecutive runs, K is the average number of communities, Q is the average moduluraty, and L is the average description length found for 10^4 runs.

Between week variation of information

The variation of information, VI, can be used to find the distance between community structures in networks with overlapping nodes [4]. Thus, this measure can be used to compare community structure

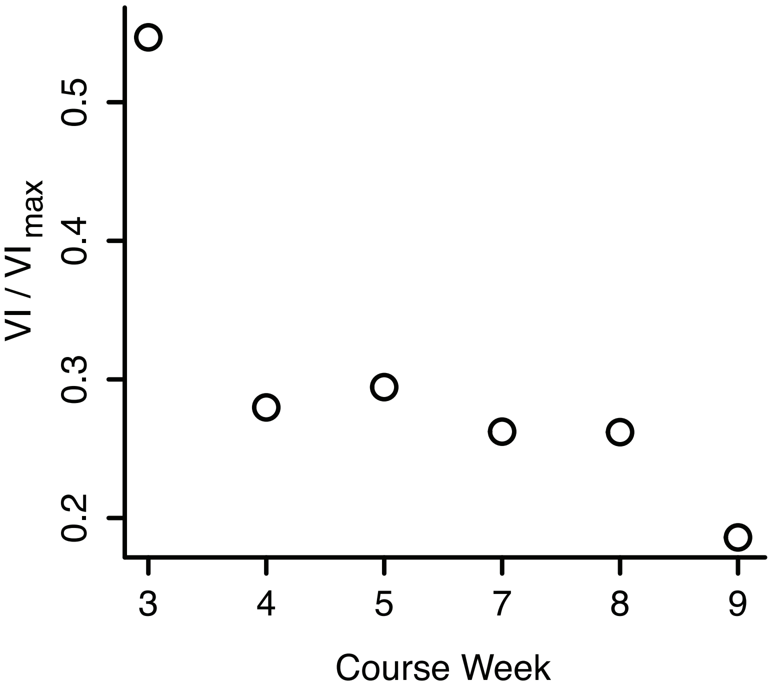

Figure S3. Between week variation of information. Each dot represents the variation of information between a week and the previous week. This is why the numbering starts from course week 3 and not 2. We used $VI_{max} = \log_2 n_{overlap}$ to normalize distances.

between weeks. For pairs of consecutive weeks we calculate VI (see Methods section in main article) between the partitionings each week with the minimum description length, $\mathcal{L}$. We normalize the found distances with $\log_2 n_{overlap}$, which is the maximum variation of information, VI_{max} between partitionings.

As can be seen in Figure S3, the distance drops over the course of the study, indicating at community structure stabilizes over time. The non-normalized distances are are 3.5, 2.0, 2.1, 1.7, 1.7, and 1.3 respectively.

Module size versus module flow rate

A community will accumulate flow rate based on both the sheer number of students in the community but also on the individual flow rates of the students. To find the extent to which community size explains accumulated flow, we made a simple linear fit of accumulated flow versus community size. As may also be gathered from Figure S4, the fit is by no means perfect, but the relation is signifcant. As a summary of the model, $\bar{R}^2 = 0.5669, p < 2.2 \cdot 10^{-16}, F = 126$. To understand more fully how structure of a community

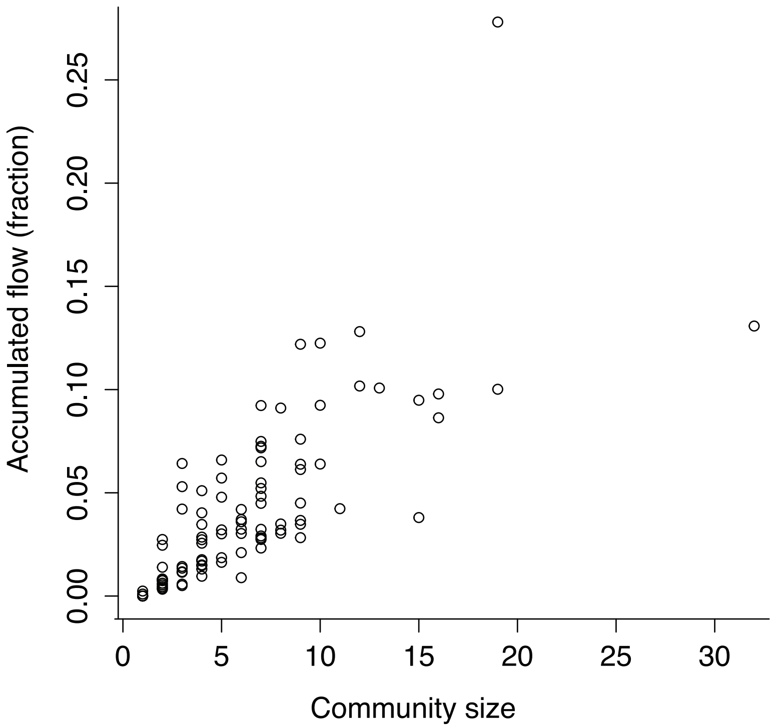

Figure S4. Plot of community size versus accumulated flow rate.

accounts for the accumulated flow, other factors need to be included.

Detailed results of segregation calculations

Tables S3-S6 show the per group Z-scores for 10^4 re-distributions and subsequent calculation of D_k's (see the Segregation measure section in main article). The score is significant, when $Z > 1.96$.

References

1. Rosvall M, Bergstrom CT (2010) Mapping change in large networks. PLoS ONE 5: e8694.

2. Reichardt J, Bornholdt S (2006) Statistical mechanics of community detection. Physical Review E 74: 016110.

3. Leicht E, Newman M (2008) Community structure in directed networks. Physical Review Letters 100: 118703.

4. Meilă M (2007) Comparing clusterings—an information based distance. Journal of Multivariate Analysis 98: 873–895.

Table S3. Course week 3 segregation.

Community	Z_{Cohort}	Z_{Cohort}	Type
1	3.7963866284	1.7385227452	c
2	0.2502614058	-0.5999679568	-
3	4.5860397119	1.6982198836	c
4	5.5563506287	1.7031356095	c
5	4.3370829175	1.2509653227	c
6	2.0647153213	-0.303785116	c
7	4.8864700176	-0.56081113	c
8	0.7333223929	-0.673683763	-
9	1.4395946406	-0.8687836047	-
10	0.4707876624	-0.723383317	-
11	3.8083330981	-0.9157366677	c
12	0.6226784735	2.3247973295	g
13	3.7960234342	-0.8421885197	c
14	2.2514079494	0.7137384453	c
15	1.9622867007	-0.5632999854	c
16	0.8943862202	-0.6825859016	-
17	-0.6295762084	-0.6712455593	-
18	-0.4318189716	-0.1250279837	-
19	0.9866694132	-0.5822771697	-
20	1.3101923449	-0.85833554	-
21	2.1119479998	0.7066433188	c
22	1.8620274082	8.653723832	g
23	0.8703785107	0.3186274207	-
24	5.2203460247	-0.9328440301	c
25	-0.413685652	-0.1208626837	-
26	1.3947367426	0.2815911595	-
27	-0.782562803	-0.1319644101	-
28	-0.405974049	-0.1282905246	-

Type indicates if and how a community is segregrated, that is if either $Z_{Cohort} > 1.96$ (c), $Z_{Cohort} > 1.96$ (g), both > 1.96 (cg) or both < 1.96 (-)

Table S4. Course week 4 segregation.

Community	Z_{Cohort}	Z_{Cohort}	Type
1	4.8754558304	-0.6861190625	c
2	3.5711431373	4.731060297	cg
3	-0.429179219	-0.5637749497	-
4	4.6425001473	-0.5060560378	c
5	0.4157401067	-0.7275233515	-
6	4.8906983822	8.5361252812	cg
7	4.3037325681	1.6485845576	c
8	6.3139198556	-0.7342353648	c
9	4.598592445	1.6073756573	c
10	1.928114553	0.7331259829	-
11	2.1261150118	-0.3477147239	c
12	1.936448378	-0.938583344	-
13	0.3057480446	-0.5569118463	-
14	4.6578425606	0.1919733758	c
15	2.3582041098	-0.2984646694	c
16	-0.6106446422	0.6699820247	-
17	3.9295073429	-0.9182165094	c
18	-0.0137346723	2.9982228714	g
19	-0.4071752972	-0.1333648753	-
20	1.6515634153	0.6607777038	-
21	0.8083989205	0.3053549656	-
22	-0.5048271303	-0.3990630874	-
23	1.4711800525	0.2901631286	-
24	-0.5652957578	0.3086792025	-
25	-0.3972179378	-0.1200879616	-
26	2.6919791414	-0.1211579842	c
27	-0.9547507757	-0.1206926067	-
28	-1.1077067884	1.8606309117	-

Type indicates if and how a community is segregrated, that is if either $Z_{Cohort} > 1.96$ (c), $Z_{Cohort} > 1.96$ (g), both > 1.96 (cg) or both < 1.96 (-)

Table S5. Course week 5 segregation.

Community	Z_{Cohort}	Z_{Cohort}	Type
1	0.7289259554	1.1720487814	-
2	5.2275855114	-0.505491634	c
3	2.8594190037	3.9139246675	cg
4	4.3879196474	1.6488657947	c
5	1.3595991271	1.1061446946	-
6	1.8286675941	-0.1744697937	-
7	1.1890715671	-0.332812553	-
8	4.4792631562	2.3813357929	cg
9	0.5720154341	0.3252341323	-
10	2.6408447039	2.7226829852	cg
11	2.9389144479	-0.5732909991	c
12	5.8446051919	-0.168879729	c
13	2.2210324398	-0.3698927798	c
14	5.6049790324	-0.7136695606	c
15	1.8950950039	0.7053383856	-
16	0.153206155	0.7013906981	-
17	3.3971224711	4.2720254002	cg
18	0.0522954928	-0.1155089263	-
19	0.0513053765	-0.110978409	-
20	-0.2771281681	-0.1123684331	-

Type indicates if and how a community is segregrated, that is if either $Z_{Cohort} > 1.96$ (c), $Z_{Cohort} > 1.96$ (g), both > 1.96 (cg) or both < 1.96 (-)

Table S6. Course week 9 segregation.

Community	Z_{Cohort}	Z_{Cohort}	Type
1	4.0696489624	-0.6914605499	c
2	2.7447948446	6.1073091363	cg
3	1.3944873531	0.5189346018	-
4	-0.1413967949	1.5107713418	-
5	3.415971134	-0.645496691	c
6	4.0028463407	1.8301336161	c
7	2.0044824771	1.3297352183	c
8	10.4380714943	-0.181288073	c
9	2.5468167318	2.5552792862	cg
10	1.0232770432	1.0941060189	-
11	1.5148586604	1.0707320995	-
12	-0.1211784232	4.2079853213	g
13	3.2224523624	-0.6736253962	c
14	1.8853398615	0.0730752136	-
15	-0.3616048145	4.1596300063	g
16	0.3692412393	0.3603600276	-
17	1.6335242432	-0.0757383444	-
18	0.3259088308	-0.5614612729	-
19	1.5867805427	-0.5645417711	-
20	-0.9175414744	-0.543113059	-
21	0.3319931378	-0.5582259976	-
22	-0.9031785743	-0.5653118031	-
23	0.3382690846	-0.5609991415	-
24	-0.9349441839	-0.5591504498	-
25	1.6009109595	-0.5546815273	-

Type indicates if and how a community is segregrated, that is if either $Z_{Cohort} > 1.96$ (c), $Z_{Cohort} > 1.96$ (g), both > 1.96 (cg) or both < 1.96 (-)
